# Motion Sensor-Based Detection of Outlier Days Supporting Continuous Health Assessment for Single Older Adults

**DOI:** 10.3390/s21186080

**Published:** 2021-09-10

**Authors:** Marc Mertens, Glen Debard, Jesse Davis, Els Devriendt, Koen Milisen, Jos Tournoy, Tom Croonenborghs, Bart Vanrumste

**Affiliations:** 1 Mobilab & Care, Thomas More University of Applied Sciences Kempen, Kleinhoefstraat 4, 2440 Geel, Belgium; glen.debard@thomasmore.be; 2 Department of Computer Science, KU Leuven, 3001 Heverlee, Belgium; jesse.davis@kuleuven.be (J.D.); tom.croonenborghs@kuleuven.be (T.C.); 3 Department of Public Health and Primary Care, Academic Centre for Nursing and Midwifery, KU Leuven, 3000 Leuven, Belgium; els.devriendt@kuleuven.be (E.D.); koen.milisen@kuleuven.be (K.M.); 4 Department of Geriatric Medicine, University Hospitals Leuven, 3000 Leuven, Belgium; jos.tournoy@uzleuven.be; 5 Department of Public Health and Primary Care, Gerontology and Geriatrics, University of Leuven, 3000 Leuven, Belgium; 6 eMedia ResearchLab and STADIUS, Department of Electrical Engineering (ESAT), KU Leuven, 3001 Heverlee, Belgium; bart.vanrumste@kuleuven.be

**Keywords:** monitoring of elderly people, PIR motion sensors, sensor network, assisted living, behavior analysis, assisted care, synthetic data

## Abstract

The aging population has resulted in interest in remote monitoring of elderly individuals’ health and well being. This paper describes a simple unsupervised monitoring system that can automatically detect if an elderly individual’s pattern of presence deviates substantially from the recent past. The proposed system uses a small set of low-cost motion sensors and analyzes the produced data to establish an individual’s typical presence pattern. Then, the algorithm uses a distance function to determine whether the individual’s observed presence for each day significantly deviates from their typical pattern. Empirically, the algorithm is validated on both synthetic data and data collected by installing our system in the residences of three older individuals. In the real-world setting, the system detected, respectively, five, four, and one deviating days in the three locations. The deviating days detected by the system could result from a health issue that requires attention. The information from the system can aid caregivers in assessing the subject’s health status and allows for a targeted intervention. Although the system can be refined, we show that otherwise hidden but relevant events (e.g., fall incident and irregular sleep patterns) are detected and reported to the caregiver.

## 1. Introduction

The aging population has motivated interest in prolonging people’s ability to live independently. Many factors, such as an individual’s mental and physical condition as well as how comfortable or safe he/she feels living alone, affect an individual’s ability to live independently [1]. Consequently, assessing an individual’s capability to live at home is a continuous process. Often, caregivers evaluate an elderly person’s independence via a questionnaire. However, this is a time-consuming process and the results do not always give an objective picture of the person’s status as answers can be interpreted subjectively by the caregiver, or the subject’s feedback can be inaccurate [2]. Furthermore, it relies on a person’s ability and willingness to give an accurate overview of his health status and report important events such as illnesses, fall incidents, and changing sleep patterns. Unfortunately, elderly individuals often forget to mention these facts during interviews [2].

Automated continuously monitoring involves collecting and analyzing data with minimal human involvement. This is an attractive option to support the elderly population’s health needs, which can be used to address several different tasks. One use is to automatically continuously and objectively collect data that may help assess an individual’s health status. Possibly, these data can even be analyzed to detect longer-term changes in a person’s behavior. A second use is to help an individual perform daily tasks, such as reminding him/her to take his/her medication [3]. A third use is to raise an alarm if an event is detected that represents an immediate threat to a person’s health or well being such as falling [4]. These examples illustrate how an automated approach can support both the elderly themselves by, for example, helping them remain self reliance (performing ADL, taking medicines, etc.) and their caregivers by, for example, promoting safety (estimating an individual’s risk of falling, detecting fall incidents, etc.). Ultimately, automated monitoring offers the potential to prolong an individual’s ability to live independently at home.

This paper describes a system that analyzes remote sensing data collected by non-intrusive, passive infrared motion detection sensors (PIR) to identify days that show locational behavior which deviates significantly from recent history. These deviations, indicating a change of habitual patterns, may be caused by the occurrence of an health-related event that needs attention. Our approach, Motion-Based Deviating Day Detector (MoBaDDD), analyzes a subject’s observed locational patterns by computing the distance between the current day’s presence pattern to a summary of the person’s recently observed presence patterns. A highly dissimilar day could indicate a condition such as being ill, having a fall incident, or a changing sleep pattern. After detecting a deviation, a statistical analysis of the underlying motion data helps the caregiver assess the subject’s status by providing clues about the cause of the deviating day through an information dashboard. An advantage of MoBaDDD is that it automatically establishes a baseline after a short period of data collection and thus does not require any person-specific background knowledge.

Our system distinguishes itself from other research in the combination of specific approaches. It is a completely self-learning, data-driven unsupervised system, unlike many other systems [5,6,7,8]. It uses only motion data from a simple motion sensor, in contrast with multimodal sensor systems [7,8,9,10,11,12,13]. Besides detection of a deviating day, it also provides the caregiver with a possible cause of the deviation unlike other research [6,8,9,11,12,14]. It also uses a general, agnostic approach which detects anomalies on a very generic level. Information as to the probable cause of the anomaly is also provided to the caregiver.

The proposed algorithm is evaluated on both simulated and real-world data. The simulated data are used to test the algorithm and to tune the initial system’s parameters. Real data were collected from the homes of three single older individuals for between three and 18 months. Solely for the purpose of validating this research, video cameras were also installed to obtain ground-truth labels and to verify detected events. From the collected data, our algorithm was able to detect deviating events such as when the subject was ill, fell, or slept in the living area as opposed to the bedroom. A clinical researcher visited each subject every two weeks for interviews and status evaluation. These observations, together with the video images, provided correlations between events detected by the algorithm and the findings from the researcher.

## 2. Related Work

Active assisted living for supporting older people is a very active area of research. Systems using sensors can monitor behavior and physiological data to monitor and detect specific health or social related issues. Examples in this domain of research are Austin et al. [15] who use sensors to detect loneliness or Marschollek et al. [16] and Mellone et al. [17] who focus on fall risk specific assessment. Other examples are detection of respiratory problems [18] and detection of urinary tract infections [19]. Next to this research domain towards specific targets, there has also a wide variety of work been done in detecting general anomalies in behavior of older persons. Our research also focuses on detection of these more general anomalies. These changes in behavior can be an early indicator of functional, health or cognitive decline of an elderly. These anomalies can be disturbances in performing activities, changing activity levels, changing mobility or a significant change from an elderly person’s regular behavior. This overview only considers research that detects anomalies using sensors integrated into a resident’s environment as this is the most closely related area to this work. Additionally, wearables deployed for this purpose are often considered to be intrusive and there is always the risk that people forget to wear them.

Within the research of detecting anomalies, different approaches exist; supervised versus unsupervised systems, multimodal and single modal systems, general agnostic anomaly detection and ADL specific based detection. Finally, it is also important to consider which information in which format is given to the caregiver.

In [6], learning is supervised by annotating activities through diary notations by the elderly. Supervised learning is also used in [7], where the subjects are asked to perform predefined tasks, and in [5], where only one appliance is used to detect anomalies. Our work is unsupervised and data-driven; only a short learning period is used to set user-specific detection thresholds.

As for multimodal and single modal systems, researches such as those in [7,9,10,11,20,21] use more than one sensor modality to detect activities and anomalies. For example, Cook et al. [9] use sensors to detect water flow, motion, temperature, burner, phone use, and others to characterize the quality of activities and detect anomalies. They constructed a labeled dataset and assessed the performance of five predefined activities. The anomalies are defined by the inconsistency of performing activities, such as difficulties in finding a telephone number, leaving the burner on, or forgetting to take medication. Activity performances are considered anomalous if the time they take to complete differs from the normal distribution by +/−2σ. The quality of performance of activities is considered an indicator for functional wellbeing. Skubic et al. [8] use a bed sensor, a temperature sensor, and motion sensors. Lundstrum et al. [11] use door, motion, as well as bed and chair occupancy sensors to model, detect, and explore short time changes in human behavior. They use a technique derived from Random Forests to detect deviating patterns in time (e.g., eating breakfast at night), in space (e.g., falling in the bathroom), and in transition (e.g., waking up at night and leaving the home. Paudel et al. [7] rely on the CASAS dataset from Washington State University to discover patterns and anomalies in the activities of residents. This dataset contains sensors for motion, temperature, item use, burner use, hot and cold water, and open or closed doors. Using a graph-based method, they detect anomalies as sign of health decline in the elderly. Nadeem et al. [21] use the built in sensors of a smartphone to classify human activity which requires the smartphone to be attached to the waist of the test person. Jain et al. [10] use the classification of activities resulting from multiple sensors deployed in a TigerPlace, such as motion sensors, bed sensors, and Microsoft Kinect sensors. While the results of these researches are promising to detect anomalies, they require a complex installation and maintenance procedure or very specific setup and calibration. Our system uses only simple, robust PIR sensors, in a meshing network which assures simple installation.

On the abstraction level of anomaly detection, there is a difference between a general, agnostic approach such as ours and an approach in which first ADL are classified. For instance, several studies focus on classifying activities before detecting anomalies, such as in [5,7,9,14]. Cook et al. [9] use five predefined activities which represent basic and complex activities from activities of daily living (ADL) to perform a quality assessment. Aramendi et al. [14] assess the functional health of elderly by first detecting activities from the instrumental activities of daily living-compensating (iADL-C) scale. Paudel et al. [7] use activity detection strongly based on iADL to detect temporal, spatial and behavioral anomalies. Furthermore, Alcala et al. [5] use one predefined activity, kettle use, to define behavior and deviation of this behavior. They learn a household individual schedule for kettle usage. Kettle usage outside the prediction model is considered an anomaly and an alert is raised. However, activities are confined to only one activity. Although activities defined in instruments such as ADL and iADL clearly indicate the functioning ability of a person, we believe that these miss some important behavioral changes such as wandering behavior or changing sleep wake patterns. Therefore, existing approaches may not detect anomalies related to these phenomena.

Another approach for detecting anomalies is to map behavior to a model or to compare current observations to recent history. The authors of [12,14] use the first rule. Mshali et al. [12] build a forecasting behavioral model to compare to a Markovian modeled dataset to detect deviations. They use activities from the Functional Autonomy Measurement System (SMAF) which is used to assess dependency. Activities used are from ADL (e.g., washing, toileting), mobility (e.g., walking), communication (e.g., speaking), and iADL (e.g., sleeping). For these activities the duration and frequency is monitored. If observed duration or frequency for performing an activity is outside the forecasted limits, it is considered an anomaly. Furthermore, Aramendi et al. [14] built several classification models to explore the possibility of detecting functional health decline.

To detect anomalies, some researches use experimentally defined thresholds. For example, Jain et al. [10] use a threshold to define a deviation if an activity is higher than an experimental defined threshold above the mean from the last two observed weeks. Novak et al. [22] use Self-Organized Maps over motion activities to detect anomalies, but this approach requires manually tuning a timing parameter to avoid false alarms. By contrast, our approach uses a threshold that is automatically set and which can adapt to an individual’s lifestyle behavior, given a small unsupervised learning period.

Finally, it is important to look at the information given to the caregiver resulting from anomalies detection. In many studies, this is long or intermediate term information such as gradual functional decline [5,9], health decline [6,12,14], or cognitive decline [7], among others. In [8], deviating days are reported to the caregiver, who has to invest sensor data to determine the root of the anomaly. Our work focuses on providing immediate information to the care giver about current observations that deviate from what is expected.

The above-mentioned researches provide an overview about how anomalies can be detected to give an indication of a person’s functional or health condition. In our research, we developed a system that informs the caregiver if in the last 24 h period a deviation has occurred using a clear semantic feedback to the caregiver. It only uses one simple sensor modality, motion sensors, which largely simplifies installation and maintenance. The system is unsupervised and can be used to monitor a person’s health status and aid older people who live alone. Several principles informed the design of our approach, and help contrast it with prior work. When developing our system, we wanted to extract health related information, assess this information, and alert the caregiver if possible health related events have occurred. To lower the acceptation threshold of technology by elderly [19], we focused on a non-intrusive and contactless monitoring framework that uses readily available and low-cost sensors. This contrasts with work that uses expensive sensors [7,9] or intrusive ones [10,11]. We aimed for a simple and reliable prediction framework that is able to automatically adapt to each person’s individual living habits without needing to extensively configure and tune the system. We analyze an individual’s behavior by considering their observed presence patterns within their living environment and detecting when they deviate from their typical pattern. Finally, we provide the caregiver with information that describes how the current observations deviate from baseline behavior. For instance, this may entail stating that the person spent significantly more time in the bedroom on a specific day than in the recent past.

## 3. Materials and Methods

### 3.1. Data Acquisition

**Figure 1:**
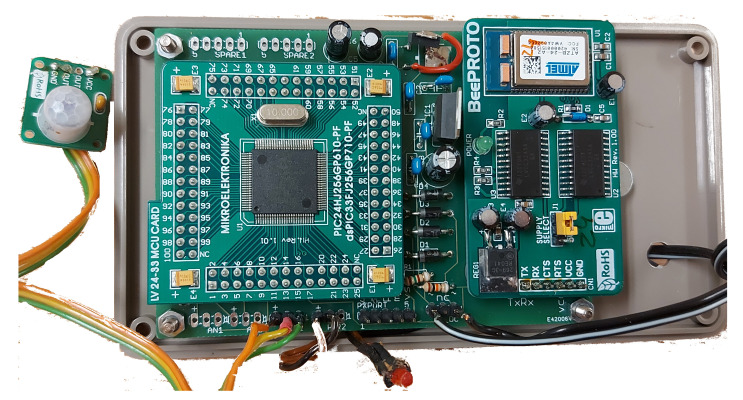
The electronics of the PIR sensor as installed in the residences.

In the proposed system, a passive infrared (PIR) motion sensor (Figure 1) is installed in every room of the residence. The sensor signal is processed by a Microchip dsPIC33F256 (Microchip Technology Inc, Chandler, Arizone, USA) microcontroller together with the timing and heartbeat mechanisms. The processor communicates through a MicroElectronics ZigBee protoboard (MikroElectronika (’Mikroe’), Beograd, Serbia) that features an ATZB-24-A2/B0 ZigBit module. When motion is detected, the sensors send data to a local computer using the wireless ZigBee protocol (IEEE802.15.4 standard). The distance from the sensor to the logging computer with the ZigBee receiver (the network coordinator) is not critical due to ZigBee’s meshing network principle: each ZigBee node can automatically relay data from other sensors towards the ZigBee controller. This ensures that data from a node that is too far away to directly communicate with the receiver will be relayed to the receiver by nodes positioned physically between it and the receiver. This property greatly simplifies installation as it makes it unnecessary for the layout of a home to enable placing the PIR sensors within close proximity to the main controller. Any commercially available PIR sensor with ZigBee could be used for the monitor system. All data are written to a time-stamped log file on the PC in sequential order.

The PIR sensors are Mikroe motion sensors boards equipped with an AMN11112 sensor (MikroElectronika (’Mikroe’), Beograd, Serbia), and their digital output is connected to an input port of the Microchip dsPIC33F256 microcontroller. In order to prevent unnecessary data flooding over the ZigBee network, each sensor is configured such that the minimum time interval between two motion detected events is three seconds. If the system does not receive a signal from a specific sensor for a long period of time, the system does not know if there has simply been no movement near the sensor or if the sensor has stopped functioning. Therefore, each sensor is configured to issue a “system heartbeat” event if it has not detected a motion event in the previous ten minutes to indicate to the monitoring system that it is still functioning.

Acquiring a complete locational behavior pattern requires installing (at least) one PIR sensor per room. It is essential to check that an installed sensor covers the complete room. If it is not possible for a single sensor to cover the room, then the room should be divided into zones with one PIR sensor for each zone. In this case, it is important to avoid that sensors have an overlapping detection area, which can be achieved by adding small panels on the side of the sensor to create a directional field of view. The zones can either be treated as one or multiple locations.

**Figure 2:**
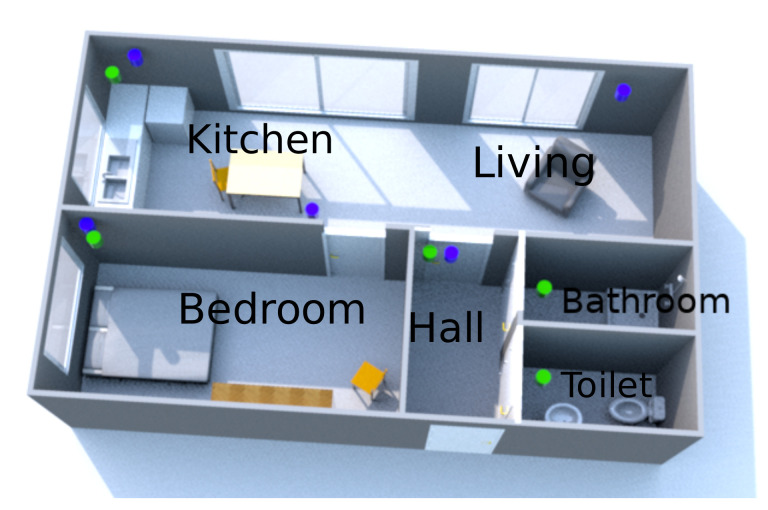
Simplified overview of location 1. The locations of the five motion sensors (green cylinders) and cameras (purple cylinders) are shown.

Figure 2 shows an illustrative home layout. Five PIR sensors are placed in the residence. The kitchen and living room are physically in one place which is monitored by one PIR sensor.

### 3.2. Representation of Raw Data

Every motion detected event is logged in the format {Unix format timestamp, loc_id, event}, where “loc_id” refers to the location where the motion was detected and the event is either “motion detected” or “system heartbeat”. System heartbeat messages are omitted from the subsequent analysis.

#### 3.2.1. Transformed Data

Each day is represented as a string loc${}_{t,d}$ of location symbols, where *t* is the interval length in seconds and *d* is the date. Starting at 00:00, day *d* is divided into non-overlapping equal-length time intervals. The user can specify the duration of the interval. Each interval is labeled with a location symbol. The label is determined by a majority vote based on the number of motion events (i.e., presence) detected in each location during the interval. If no motion is detected during an interval, the label of the last known location is assigned to that interval. This is a fast and simple way to assign a location label to an interval which gives a reasonable measure of presence, particularly if the interval is sufficiently small (e.g., 60 s). Therefore, for each time interval, an individual’s presence is allocated to one specific location by majority vote of motion detections.

Referring to the example location of Figure 2, the following labels are used: “B” for bedroom, “A” for bathroom, “T” for toilet, “H” for hallway, “L” for living room/kitchen, and “O” for outdoor. In this layout, the kitchen and living room are physically in one place which can be covered by one sensor. The following string shows a hypothetical day with a one hour time interval:

$${\mathrm{loc}}_{3600.2}=“\mathrm{BBBBLTLLLLTLLBBOOBBBBBBB}”.$$

**Figure 3:**
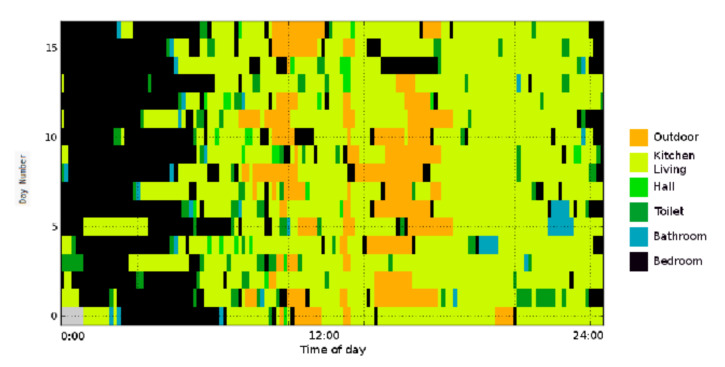
An example of a heat map illustrating several days of an individual’s locational history over the course of a day. For visualization purposes, each day is divided into 48 30-min slots (in reality, time slots would be for instance 60 s). Each color represents a different location.

This indicates that on the second day, the person was in his bedroom until 4 a.m., spent most of the next hour in the kitchen/living room, then went to the toilet, etc. As shown in Figure 3, these patterns can be visualized using a heat map where each location corresponds to a color.

#### 3.2.2. Real-Life Data from a Small-n Case Study

A small-n case study, which is typical for behavioral analysis research, was performed to validate the algorithm. The study aims to address whether the MoBaDDD algorithm can detect relevant deviating days. The case studies involved collecting data about three different subjects. Each subject lived in a different location and lived alone as this was the targeted subject group. One subject was monitored for 18 months, and the other two for three months. At each location, the monitoring system was installed, with one motion sensor in each room. An example of an installation is shown in Figure 2. Table 1 gives an overview of the different rooms monitored in each location. All rooms but the toilet and bathroom were equipped with one or two cameras. The video data were used to validate the monitoring system’s observations.

It is not always possible to install a sensor outdoors, e.g., in the hallway of a residential setting because other residents that pass by the door would falsely trigger the sensor. Therefore, a person is considered to be outdoors if the last detected activity was in a location that unambiguously leads outside and no new activity has been detected for at least five minutes. Neither location 2 nor 3 meets this criteria. In these locations, outdoors is not considered. Furthermore, in locations 2 and 3, the toilet is the same room as the bathroom, which makes toilet visits more difficult to discern.

The entire study was conducted with the approval of the medical ethics committee of KU Leuven and the study participants (ethical file S53549). A clinical researcher evaluated each subject using a variety of tests at the start, end, and, if applicable, every three months during the study period to assess the evolution of each subject’s physical and cognitive condition. Specifically, the following tests were administered: the TGUG (Timed-get-up-and-go) [23], Katz ADL (Katz Activities of Daily Living) [24], IADL (Instrumental Activities of Daily Living) [25], MMSE (Mini Mental State Examination) [26], MOCA (Montreal Cognitive Assessment) [27], GDS (Geriatric Depression Scale) [28], DI (Delirium Index) [29], and CAM (Confusion Assessment Method) [30]. Table 2 gives an overview of the test results for each subject.

### 3.3. The MoBaDDD Algorithm

**Figure 4:**
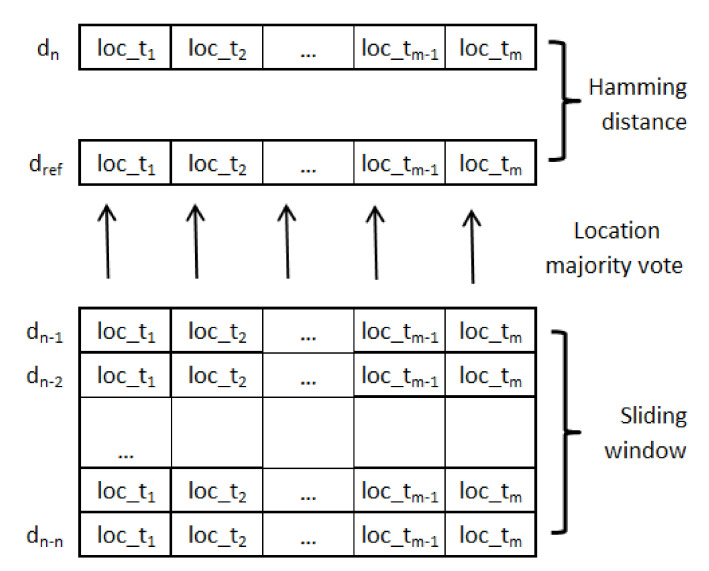
Overview of the MoBaDDD algorithm. Each day is divided into *m* time intervals. The current day’s (*d*${}_{n}$) reference day *d*${}_{ref}$ is constructed determining each time interval’s location by a majority vote over the preceding *n* days. Finally, the Hamming distance is calculated between *d*${}_{n}$ and *d*${}_{ref}$.

The MoBaDDD (MOtion-Based Deviating Day Detector) algorithm analyzes motion data to detect if an individual’s presence pattern deviates from recent typical behavior. It focuses on short trending changes, that is, days that stand out as being different compared to recent history. Algorithm 1 provides a detailed overview of MoBaDDD, and Figure 4 illustrates how it works. At the beginning of each day, a reference day is constructed to capture a subject’s recent, typical presence pattern. At the end of the day, the distance between the current day and its reference day is calculated. If the distance exceeds a threshold that is automatically computed via a statistical formula, the algorithm considers the current day to be a deviating day and issues an alert. After detecting a deviating day, the system can analyze the collected statistics to propose possible reasons for the deviation (e.g., significantly more toilet visits, unexpectedly long time spent in bathroom, etc.). The caregiver can use these informational clues during the conversation with the subject to assess the status and to find the root cause of the deviating day.

#### 3.3.1. Reference Day

A reference day is described by the same number of non-overlapping, equal-length time intervals as the transformed data. A label is assigned to each time interval in the reference day by conducting a majority vote over this interval’s labels from the previous *n* days. The value of *n*, which is a user-defined parameter, influences the efficacy of detecting gradual or sudden (i.e., one day) changes, and it largely depends on the person’s specific behavior pattern.
**Algorithm 1** Overview of the MoBaDDD algorithm**Require:***n*: length sliding window; τ: time interval; ϵ: threshold $d_{i}\leftarrow$ current day
 **repeat**  *//Build reference day by majority vote per time interval*
  **for**
i=0 to 24*60*60/τ
**do**
   $d_{ref}.i\leftarrow mode\left(days.i\right)$
*//d.i is d’s $i^{th}$ time interval*
  **end for**
  $dist\leftarrow d_{h}\left(d_{i},d_{ref}\right)$
*//See Equation (1)*
  ϵ←
*Compute via Equation (2)*
  **if**
dist>ϵ
**then**
   set $d_{i}$ as deviating day
  **end if**
  $d_{i}\leftarrow d_{i+1}$
 **until** end


#### 3.3.2. Detecting Deviating Days

If a day differs significantly from its reference day, this could indicate a deviation from the subject’s regular behavior arising from a health condition on that day. To measure the difference, we compute the normalized Hamming distance between the string representations of the day (*a*) and its reference day (*b*):

(1) $$d_{H}\left(a,b\right)=\frac{1}{m}\times \sum_{i}^{l}a_{i}\neq b_{i}$$

where m=24*60*60/τ is the chosen number of time intervals used to represent each day. This simply divides the number of positions where the symbols in the two strings differ by the length of the strings. This work only compares equal length strings.

The number of time intervals *m*, depends on the chosen time interval. If, for instance, the time intervals are chosen to be 60 s, *m* would be 1440 (60 s × 24 h = 1440).

The day is considered to be an outlier if the distance is above a threshold ϵ, which is defined as

(2) $$\epsilon \left(i,n,\rho \right)=d_{H}\bar{(i},n)+\rho s_{d_{H}\left(i,n\right)}$$

where *i* is a certain day, *n* number of days looked back, and ρ is a tunable parameter. Essentially, a day is an outlier if its distance to the reference day is greater than the average Hamming distance over the *n* preceding days plus ρ times the Hamming distance’s standard deviation over the same period. Normally ρ = 3, which is based on the standard 3-sigma rule, but we can fine-tune the system by selecting a person-dependent value for ρ. This statistical moments model offers two advantages over a model with strict limits: (1) no prior person-specific knowledge is required and (2) the limits are defined based on observed user behavior.

#### 3.3.3. Documenting a Deviating Day

If a deviating day is detected, the system provides the caregiver with detailed statistical information. Important indicators include the time spent in a location or how many times a certain location is visited. For example, a sudden increase in the number of toilet visits can indicate a health-related issue, such as being ill, a urinary tract infection, etc.

Again, a statistical moments model is used to suggest which indicators might explain why a day was considered to be deviating. For the *n* days prior to the deviating day, we compute the mean and standard deviation for both the time spent in each room and the number of visits to a room per day. The system compares the time spent in and number of visits to each location on the deviating day to their respective means plus/minus their standard deviation. If either of these parameters exceed this threshold, then this information is reported.

**Figure 5:**
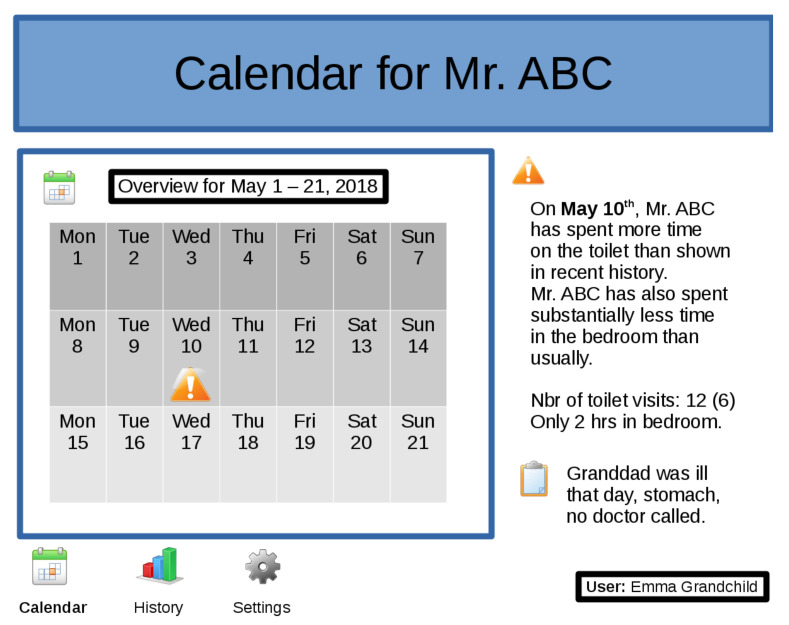
Example of a dashboard with a possible relevant event. The calendar gives an overview while some possible causes are given on the right side, together with the possibility to add a quick note.

The report takes the form of a clue to the caregiver such as “More time was spent in the bathroom than expected” or “Fewer toilet visits than expected”. This information can be visualized to the caregiver using a dashboard as depicted in Figure 5. The feedback can point to possible explanations for the deviation. However, the final interpretation rests with the caregiver. Based on the information suggested by the system and his/her own expertise, he/she can evaluate the situation, for instance, with focused questioning of the subject. As an example, when the system suggests that the toilet is visited more often than expected, the caregiver could suspect that the subject has experienced health issues such as sickness. The suggestions can also aid as a guide when interviewing the subject.

### 3.4. Algorithm Validation with Synthetic Data

Validating algorithms such as the MoBaDDD algorithm and exploring its parameters require sufficient data. As the real-world study has data about three subjects, we generate synthetic data to aid in evaluating and selecting the values for the system’s parameters. Furthermore, the real dataset, as captured during research, is an asymmetric dataset with very few real-life adverse events. Generating synthetic data allows for injecting many adverse events at predefined moments to evaluate the algorithm and fine tune the detection parameters.

#### 3.4.1. Constructing Synthetic Data

One way to create synthetic data is to use a single, generative probabilistic model. However, given that behavioral patterns, and thus the threshold for identifying deviating days, are person-specific, using such a model is likely not ideal. Other method are successfully researched and applied, such as the hidden Markov and regression-based synthetic data generation of Dahmen and Cooke [31]. In our research, we construct person-specific synthetic data sets based on the data collected at each real-world location. This way, the specificity is reflected in our simulation data. One synthetic data set of 730 days is generated for each real-world location using the following three-step approach.

Step 1: For each time interval in the day, define the probability for detecting presence in each location. For each location, one multinomial discrete probability distribution is constructed for each time interval. For a given interval, the probability a person is in a location is simply the number of days he was in that location during the interval divided by the number of monitored days. A day is represented by using the same number of intervals as MoBaDDD will use.

**Figure 6:**
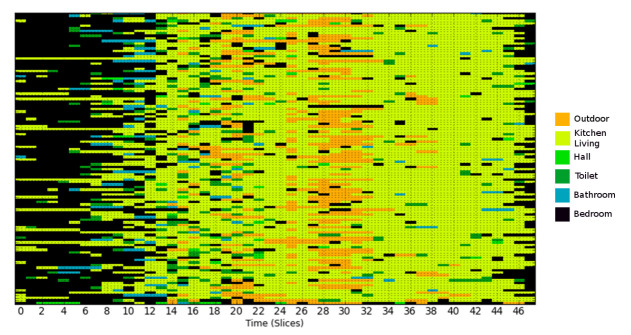
Location heat map for real data with a 30-min resolution. This low resolution is for visualization purposes; normally, resolution is much higher (e.g., 60 s).

**Figure 7:**
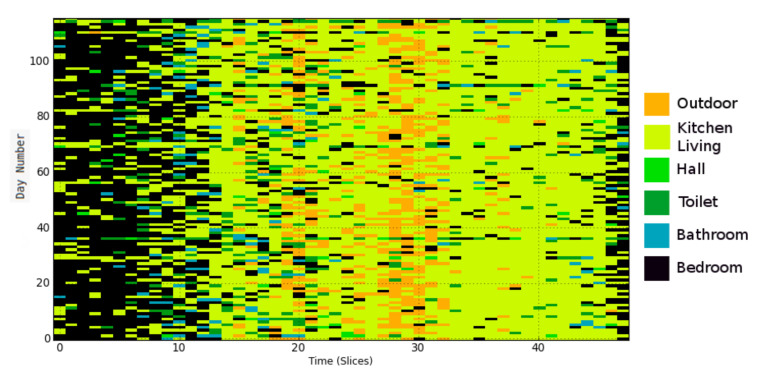
Location heat map for synthetic data with a 30-min resolution. This low resolution is for visualization purposes; normally, resolution is much higher (e.g., 60 s).

Step 2: Sample a given number of days from the probability distribution. A data set is constructed by sampling a specified number of days from the calculated location probabilities, which are used as an arbitrary discrete distribution. A single day is built by randomly sampling a label for each time interval in a day from the corresponding distribution. The data set contains similar behavioral patterns to those observed in the real data as shown by comparing Figure 6 and Figure 7.

Step 3: Insert deviating days. Deviating days are inserted at randomly spread positions. Two consecutive days cannot both be deviating days, as it is more important to detect the first deviation. A deviating day is constructed by selecting a time interval and increasing the probability of presence in one location. The perturbations are minor so that there is only a small difference between the deviating and normal days. Thus the deviation is not so obvious that it is trivial to detect. Modeling a deviating day in this way is based on the observations that, for instance, sickness or fall incidents lead to immobilization in predominantly one location. For example, when sick, a person typically resides in bed more than on a typical day. As it is impossible to model every possible deviation, this heuristic provides a good start for evaluating the algorithm. Twenty deviating days are inserted at predefined positions (days 53, 67, 79, 92, 123, 175, 188, 201, 231, 243, 271, 289, 302, 331, 412, 501, 555, 603, 673, and 701) into each data set.

#### 3.4.2. Defining the Best Parameter Set

To evaluate performance on the synthetic data, we use precision–recall (PR) plots and the F${}_{\beta }$-measure. The precision or positive predictive value (PPV) is simply the proportion of days flagged as deviating that are truly anomalous, and it is defined as

(3) $$Precision\;=\;PPV\;=\;\frac{TP}{TP+FP}$$

where TP is the number of true positives (i.e., truly deviating days) and FP is the number of false positives (i.e., incorrectly identified deviating days). The recall or sensitivity describes the fraction of detected deviating days compared to all existing deviating days:

(4) $$Recall\;=\;Sensitivity\;=\;\frac{TP}{TP+FN}$$

where FN is the number of false negatives (i.e., deviating days incorrectly classified as normal). The F${}_{\beta }$-measure combines precision and recall:

(5) $$F_{\beta }\;=\;\left(1+\beta^{2}\right)\;\times \;\frac{precision\;\times \;recall}{(\beta^{2}\;\times \;precision)\;+\;recall}$$

where β controls the trade-off between recall and precision.

The goal of the synthetic data experiments is to select the best parameter values for the sliding window size *n*, the time interval length τ, and the threshold parameter ρ. We consider different values of β for the $F_{\beta }$-measure. Typically, β = 1, which gives equal weight to precision and recall, effectively weights the cost of a FP and FN equally. However, in our situation, the cost of a FP is lower than the cost of a FN, as no immediate intervention is required. Investigating the underlying data or a simple and short interview can exclude a false positive raised by the system. On the other hand, missing a truly relevant event could result in an individual’s health status deteriorating or, even worse, missing an acute incident, such as a fall. Therefore, higher recall is desirable. However, it is also important to limit the number of FPs, because they generate an unnecessary load on the caregiver. Thus, tuning the parameters to a person-specific reporting of events is essential.

Initially setting β to 5 is a good starting choice for optimizing towards true positives for all locations. Smaller values for β reduce both the number of FPs and TPs, but recall is more important in this application. Because the results are on a sampled data set, we sample ten data sets for each location and find that setting β to 5 or 10 consistently yields good results in terms of TPs. Table 3 gives an overview of β and corresponding parameters for the three locations.

As can be seen from Table 3, β is 5 for the first two locations. For the third location, we choose β = 2, because there were too many FPs. This shows that, to achieve the best performance, it is important for the user to be able to tune the system.

## 4. Results for the Small-n Case Study

To test the MoBaDDD algorithm, a small-n case study (n = 3) has been performed on the real-life data collected from the three previously described locations. Using these data, the following three questions are evaluated:

1. How do the different choices of β in the $F_{\beta }$-measure reflect usability in a real-life setting?
2. What is the algorithm detection capability for the chosen parameter set *n*, τ?
3. How do the statistical model’s suggestions about the underlying cause of the deviating day compare to observations from the corresponding video data?

### 4.1. Case Study Method

Based on the analysis in Section 3.4.2, we use the parameters according to Table 3. The analysis begins after 2n day as the first *n* days are used to calculate statistical data to define the mean and standard deviation of the Hamming distance, and the second *n* days are used to construct the reference day. Every detected deviating day is verified by manually inspecting the video data and checking the clinical researcher’s observational reports from the visits to the subjects. Note that video cannot reveal missed events (FNs) by itself, and therefore we rely on interviews with the subjects to identify possible FNs.

### 4.2. Case Study Results

Table 4 shows an overview of the detected days that deviate from recently observed behavior for all three locations.

Next, we discuss the results for each location in detail.

**Figure 8:**
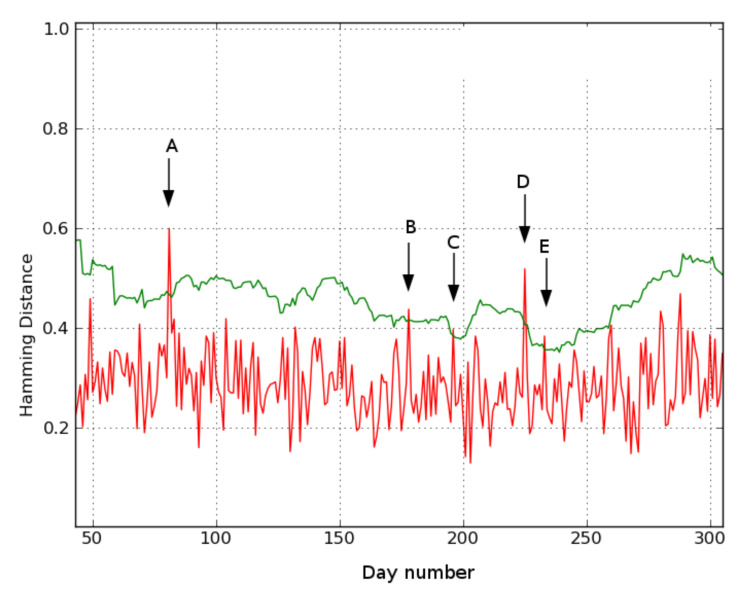
Location 1: Each day’s Hamming distance compared to its reference day using n=21 days and τ = 60 s. The green line is the threshold calculated by Equation (2). Distances exceeding the threshold are indicated by an arrow and represent a deviation (green line is calculated threshold, red line is hamming distance) ‘A’, ‘B’, ‘C’, ‘D’, ‘E’ indicate detected deviating days.

Location 1. Figure 8 shows the results for location 1. Days 81 (A), 178 (B), 196 (C), 225 (D), and 233 (E) exceed the threshold and are flagged by the system as deviations.

Days A and B show a deviating behavior. On day A, the video and underlying presence data reveal that the subject does not go to his bedroom that night. Instead, from 11:50 p.m. on the previous day, he sits at the table in his living room, falls asleep, and remains there until 5:30 in the morning. He then gets up and resumes his daily activities without having slept in his bed. Similarly, on day B, the person again does not go to his bedroom at night and instead falls asleep sitting at his living room table. On Day D, the underlying data reveal that a sensor error leads to the deviation. Although this is not of clinical relevance, it is an important detection from the perspective of monitoring the system’s status. In a later version of the system, this type of error is automatically reported via the heartbeat mechanism. On days C and E, no obvious deviating behavior appears in either the videos or the heat maps. These days can be considered false positives.

**Figure 9:**
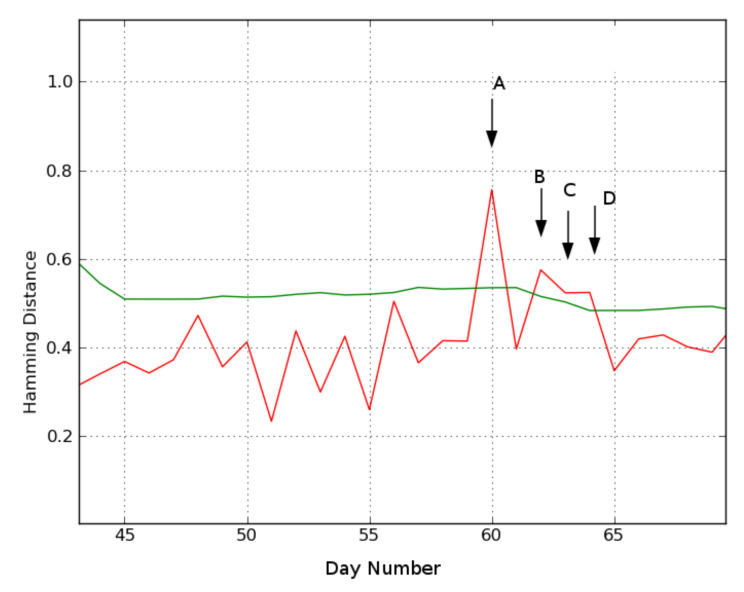
Location 2. Each day’s Hamming distance compared to its reference day using n=21 days and τ = 60 s. The green line is the threshold calculated by Equation (2). Distances exceeding the threshold are indicated by an arrow and represent a deviation (green line is calculated threshold, red line is hamming distance). ‘A’, ‘B’, ‘C’, ‘D’ indicate detected deviating days.

Location 2. Figure 9 shows the results for location 2, where days 60 (A), 62 (B), 63 (C), and 64 (D) are identified as deviating.

The video data for day A reveal that the subject is in the bathroom from 7:45 p.m. the previous day until 2:30 a.m. Then she gets ready for bed, but instead of going to bed, lies in a chair in the living room. As no cameras are installed in the bathroom, it is impossible to deduce what activities were performed. However, the length of the bathroom stay is exceptional and therefore possibly relevant to a caregiver.

Day B is flagged as another deviating behavior by the MoBaDDD algorithm. Video assessment reveals that the subject naps in the bedroom in the afternoon. After napping, she wanders around, takes some medicine, looses her balance and eventually falls in the bathroom. Then, she crawls towards her bedroom where she remains on the ground for several hours. She pulls some sheets from the bed to cover her and she stays in that position until she is found during the night by a nurse.

Day C differs from recent behavior because the person spends most of her time in the bedroom. A caregiver can use this information and the information of the previous day’s fall incident to further assess her health condition.

Finally, on day D the subject spends significantly more time than usual in the bathroom during the day and she spends time in the living room during the night.

**Figure 10:**
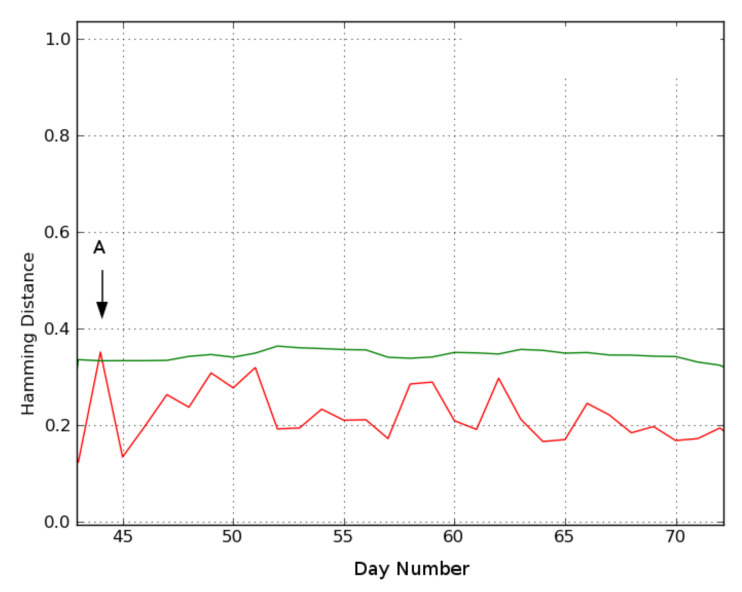
Location 3. Each day’s Hamming distance compared to its reference day using n=21 days and τ = 60 s. The green line is the threshold calculated by Equation (2). Distances exceeding the threshold are indicated by an arrow and represent a deviation (green line is calculated threshold, red line is hamming distance). ‘A’ indicates the detected deviating day.

Location 3. Finally, Figure 10 shows the results for location 3, where only day 44 (A) is considered deviating. Day A’s video reveals that the subject leaves her residence in the afternoon for a substantially longer period than in her recent history, which is not strictly an indication of any abnormal behavior.

### 4.3. Results from Comparison of Visual Conclusions with Statistically Generated Clues

The deviating days found at all three locations were analyzed by applying the statistical method from Section 3.3.3 to the underlying presence data. Table 5 compares the findings from visually inspecting the video data to the statistical method’s output. Recall that the statistical moments model merely provides clues, not all of which are conclusive. However, they mostly agree with the findings from analysis of the video data. For example, on day A in location 1 the subject did not go to his bedroom that night. Here, the system suggested less time was spent in the bedroom and more time was spent in the living room than was expected. These hints support the conclusion from the video data. On day B at location 1, the system suggested that more time was spent in the toilet while this was not observed otherwise. This could give a caregiver another anchor when interviewing the subject.

## 5. Discussion

In this paper, a system is proposed to assist in assessing longitudinally and continuously the health condition of older people living alone. It does this by providing behavior related information otherwise unknown to the caregiver. An ideal system for this task should have several characteristics:

1. Be low-cost so it is widely applicable;
2. Be easy to install in a home (i.e., does not require significant renovation);
3. Be non-intrusive and not rely on the subject to wear or use special equipment;
4. Generate a limited number of false positives;
5. Respect the privacy of the subject.

This paper details our work in developing such a system. It relies on using simple motion detector data.

Having a continuous monitoring presence patterns provides a means to detect days that stand out compared to recently observed history. The information obtained can provide valuable input to a caregiver who no longer has to rely on possibly subjective and incomplete information given by an elderly individual. The system will detect incidental behavioral changes on a day-to-day basis. As every person has a very specific behavioral pattern, the system automatically adapts to the circadian rhythm of the person. Consequently, the system does not require a tedious and detailed configuration for each installation. Detecting deviating events is typically done at the end of each day, but the algorithm allows for analyzing the current day earlier on, e.g., in the morning or at noon. Slowly evolving or gradual changes are not detected by our system and they would require other algorithmic design choices.

The proposed algorithm has been tested in a case study where three people were monitored. In all three locations, days were detected with possible relevant clinical information such as fall incidents, possible illness, and a disturbed sleep–wake pattern. Some of these were not reported by the subjects, which highlights the value of continuous monitoring. In location 1, two days were detected where the subject did not sleep in his bedroom, which may indicate a disturbed sleep–wake balance. This was not reported by the subject, but can be a valuable piece of information for a caregiver, who can investigate the cause of this behavior. A similar behavior is detected in location 2 on day A. Knowing that the subject does not sleep in the bedroom enables the caregiver to take preventive actions to help the subject.

The fall incident at location 2 on day B is clearly clinically relevant. Furthermore, the sensor data, e.g., in the heatmap, show that several hours elapsed between the fall and when the subject was found. This information would be missing if the subject does not report it. Two days following the fall incident, the subject spent most of the day in the bedroom (day C) or in the bathroom (day D). If given this information, the caregiver can check if it is related to the fall incident on day A by, for instance, specific interviewing.

Furthermore, to aid the caregiver in his assessment, a statistical evaluation method for the underlying data is proposed to help find the cause of the deviating day. The caregiver (e.g., nurse, general practitioner, or family) can decide if the provided information has clinical relevance by using the system’s clues when talking with the monitored person. As illustrated on day B for location 1, the system suggested that the person spent more time in the toilet than in previous days. However, it is not yet robust enough to conclusively indicate the root cause of the change in behavior. The caregiver can use the system’s suggestions to augment his own expertise to reach a conclusion. In the future, a more elaborate system can be developed based on these findings.

An important advantage of MoBaDD is its self-learned and adaptive threshold (Equation (2)). The threshold is set only based on a short learning period (i.e., no other person-specific information is needed), with results indicating around two or three weeks of data is sufficient. In this research, appropriate thresholds were selected using synthetic data that was generated from a model based on the characteristics of real-world data. Our automatically learned and updated thresholds would be highly beneficial for a large-scale installation of such a monitoring system.

**Figure 11:**
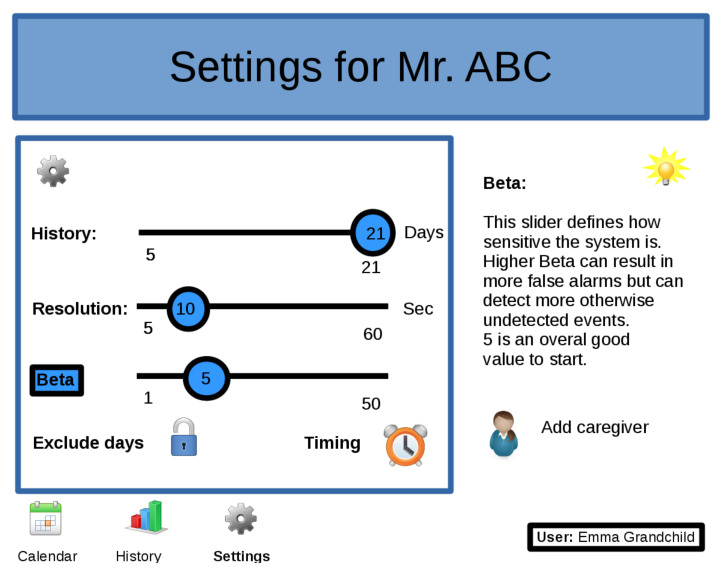
Example of a dashboard settings page. Starting from default settings, the user can easily fine-tune detection levels, exclude certain days and define the times when analysis should be done.

Still, it is possible to fine-tune the system individually with respect to reported events, which provides an opportunity to balance false positives against the risk of missing important events. For example, a deployed version could include a dashboard, as in Figure 5, to give feedback, such as recent or historical overviews, in a user-friendly way to the caregiver. It would also be possible to allow a caregiver to tune the system’s parameters via a setup page as shown in Figure 11. A self annotation mechanism, active during the learning phase of the system, could help to easily distinguish between real events and false positives. The user can also define when the system should do the analysis (e.g., at the end of the day or at midday) and which days should be excluded for analysis (e.g., weekend days, day when a trip is planned, etc.).

However, the system has some limitations. It is not guaranteed to detect all days that contain health or otherwise important events. Monitoring is limited to a person who is living alone without any free-running pets. Visits are difficult to automatically detect from the available data. Future research will address these issues. Furthermore, the specificity of the statistical method to assist the caregiver in his conclusions could be increased. On the other hand, the system can naturally capture seasonal changes. It also has the potential to account for weekly activities by constructing a day-specific reference day (e.g., compare Wednesdays to a reference day built only from recent Wednesdays). As this study only involved three participants, more clinical evidence can be collected on a more elaborate study. Furthermore, we intentionally limited ourselves to one sensor type. Other sensor types (e.g., door sensors, bed presence sensors, etc.) could give a more detailed picture of the behavior and could be explored in future work. Table 6 shows an overview of the technical characteristics, advantages, and disadvantages of the developed system.

Finally, concerning the privacy of the subject, as Meingast et al. discuss in [32], sensor networks and monitoring systems such as these can improve healthcare, both on a quality as a cost of care level. However, privacy and security concerns should be dealt with. In the implementation of systems like these, utter care should be given to issues like ownership of data, can the user control who gets to see which information, are the communication layers securely implemented, and what sort of data should be stored and where.

### Resumptive Comparative Analysis

As discussed in Section 1 and Section 2, a lot of research has been done on active assisted living for supporting older people. In this section, we present a short synthesis of systems mentioned in those sections, in comparison with our system. We consider here three grounds of contrast, as we consider them to be essential for a monitor system given the characteristics as described in Section 5: technical implementation, the abstraction level of anomaly detection, and the information provided related to the deviating event.

1. Technical implementation
  Research such as that in [7,9,10,11,20,21] uses more than one sensor modality to detect activities and anomalies. For example, sensors to detect water flow, motion, temperature, burner, phone use, and bed sensors, whereas our research focuses on one simple low-cost sensor type: PIR motion sensors. The works in [5,6,7] are based on a supervised system, while our system is unsupervised data-driven.
2. Abstraction level of anomaly detection
  Our system uses a general agnostic approach that shows locational behavior which deviates significantly from recent history, while other research firstly classifies e.g., (i)ADL and defines anomalies based on those classifications, as in [5,7,9,14], or map functional behavior to a model as in [12,14].
3. Information provided related to the deviating event
  Many researches provide long- or intermediate-term information such as gradual decline [5,6,7,9,12,14] or indicate only an anomaly where the caregiver has to interpret the raw data [8], whereas our work focuses on providing immediate information to the care giver about current observations that deviate from what is expected.

## 6. Conclusions

The aging population has motivated interest in supporting people to live independently at home for as long as possible. This research presents an approach for unsupervised automatic continuous monitoring that can provide otherwise hidden information to the caregiver. This knowledge can help to assess the health and self-reliance status of individuals living alone and provide the opportunity to assist them living in their own environment.

## Figures and Tables

**Table 1:** An overview of the different rooms present at each location.

	Loc 1	Loc 2	Loc 3
Bedroom (B)	yes	yes	yes
Bathroom (A)	yes	yes	yes
Seperate Toilet (T)	yes		
Hall (H)	yes		
Living room (L)	yes	yes	yes
Kitchen (K)		yes	yes
Outdoor (O)	yes		

**Table 2:** An overview of the subjects and the scores of each administered clinical test at the start of the monitoring (TGUG (Timed-get-up-and-go) [23], Katz ADL (Katz Activities of Daily Living) [24], IADL (Instrumental Activities of Daily Living) [25], MMSE (Mini Mental State Examination) [22], MOCA (Montreal Cognitive Assessment) [27], GDS (Geriatric Depression Scale) [28], DI (Delirium Index) [29], and CAM (Confusion Assessment Method) [30]).

	Loc 1	Loc 2	Loc 3
Monitoring period (months)	18	3	3
Sex	male	female	female
Age	75	75	79
Residence	Apartment	Service flat	Service flat
Rooms/zones	6	4	4
TGUG (sec)	11	16	13
ADL (max 24)	6	7	6
I-ADL (max 32)	14	13	8
MMSE (max 30)	21	28	30
MoCa (max 30)	17	20	26
CAM (delirium)	no	no	no
GDS (max 10)	3	6	2

**Table 3:** Overview of chosen β and resulting parameters for each location.

Location	β	ρ	n (Days)
1	5	2.8	21
2	5	2.0	21
3	2	2.5	14

**Table 4:** Summary of the results for the real-world data. No FN have been reported while interviewing the subjects.

Location	β	Nbr of Days	Poss. Relevant Days (TP)	False Pos. (FP)
1	5	305	2	2 (+1 sensor error)
2	5	72	4	0
3	5	72	1	0

**Table 5:** This table compares the system’s clues about possible reasons for a deviating day to observations derived from watching the video. For each location, ‘>’ means more than expected, ‘<’ means less than expected, and an empty field indicates no deviation.

	Observations from Video	Clues from Systems’s Statistical Model									
		**Time Spent in Room**					**Number of Visits to Room**				
**Day @ Loc**		**Bed**	**Bath**	**Toilet**	**Living**	**Out**	**Bed**	**Bath**	**Toilet**	**Living**	**Out**
Location 1											
Day A	not in bedroom at night	<			>	>					
Day B	not in bedroom at night	<		>	>	>	<	<			
Day C	no peculiar behavior observed	<	>				>				
Day D	sensor error	>	>	<	<	>					<
Day E	no peculiar behavior observed	>	>	>	<	>	<				<
Location 2											
Day A	long time in bathroom	<	>		<			>		>	
Day B	sleep in afternoon, wanders, fall	>			<		>				
Day C	long in bedroom	>			<			<		<	
Day D	long in bathroom		>								
Location 3											
Day A	out in afternoon	<	<		<						

**Table 6:** This tables gives an overview of the system’s major technical characteristics, advantages, and disadvantages.

Technical characteristics	- Simple, single modal sensors (PIR motion sensors)
	- ZigBee data communication protocol
	- Presence based behavior observation
	- Hamming Distance based anomaly detection
Advantages	- Low cost
	- Simple to install and configure
	- Self learning without the need for prior knowledge
	- Unsupervised
	- Possibility to fine-tune
	- Deviations documented with statistically relevant information
Disadvantages	- No guarantee to detect every health related issue
	- Only single elderly, living alone without free-running pets
	- Difficult to detect visitors

## Data Availability

The data supporting the conclusions of this manuscript are not available given the stipulations in the Medical Ethical file and Informed Consent documents (ethical file S53549).

## References

[1] Mahmood A., Yamamoto T., Lee M., Steggell C. (2008). Perceptions and use of gerotechnology: Implications for aging in place. J. Hous. Elder..

[2] Warren C.A.B., Williams K.N. (2008). Interviewing elderly residents in assisted living. Qual. Sociol..

[3] Pollack M.E., McCarthy C.E., Ramakrishnan S., Tsamardinos I., Brown L., Carrion S., Colbry D., Orosz C., Peintner B. Autominder: A planning, monitoring, and reminding assistive agent. Proceedings of the 7th International Conference on Intelligent Autonomous Systems.

[4] Kim J.T., Soh J.Y., Kim S.H., Chung K.Y. Emergency situation alarm system motion using tracking of people like elderly live alone. Proceedings of the 2013 International Conference on Information Science and Applications (ICISA).

[5] Alcalá J., Parson O., Rogers A. Detecting Anomalies in Activities of Daily Living of Elderly Residents via Energy Disaggregation and Cox Processes. Proceedings of the 2nd ACM International Conference on Embedded Systems for Energy-Efficient Built Environments.

[6] Eldib M., Deboeverie F., Philips W., Aghajan H. (2016). Behavior analysis for elderly care using a network of low-resolution visual sensors. J. Electron. Imaging.

[7] Paudel R. Anomaly Detection of Elderly Patient Activities in Smart Homes using a Graph-Based Approach. Proceedings of the 2018 International Conference on Data Science.

[8] Skubic M., Guevara R.D., Rantz M. (2015). Automated Health Alerts Using In-Home Sensor Data for Embedded Health Assessment. IEEE J. Transl. Eng. Health Med..

[9] Cook D.J., Schmitter-Edgecombe M. (2009). Assessing the quality of activities in a smart environment. Methods Inf. Med..

[10] Jain A., Keller J.M. Textual summarization of events leading to health alerts. Proceedings of the 2015 37th Annual International Conference of the IEEE Engineering in Medicine and Biology Society (EMBC).

[11] Lundström J., Järpe E., Verikas A. (2016). Detecting and exploring deviating behaviour of smart home residents. Expert Syst. Appl..

[12] Mshali H., Lemlouma T., Magoni D. (2018). Adaptive monitoring system for e-health smart homes. Pervasive Mob. Comput..

[13] Jakkula V.R., Cook D.J., Crandall A.S. Temporal pattern discovery for anomaly detection in a smart home. Proceedings of the 3rd IET International Conference on Intelligent Environments.

[14] Aramendi A.A., Weakley A., Goenaga A.A., Schmitter-Edgecombe M., Cook D.J. (2018). Automatic assessment of functional health decline in older adults based on smart home data. J. Biomed. Inform..

[15] Austin J., Dodge H., Riley T., Jacobs P., Thielke S., Kaye J. (2016). A Smart-Home System to Unobtrusively and Continuously Assess Loneliness in Older Adults. IEEE J. Transl. Eng. Health Med..

[16] Marschollek M., Rehwald A., Wolf K.H., Gietzelt M., Nemitz G., Meyer zu Schwabedissen H., Haux R. (2011). Sensor-based Fall Risk Assessment—An Expert ‘to go’. Methods Inf. Med..

[17] Mellone S., Tacconi C., Schwickert L., Klenk J., Becker C., Chiari L. (2012). Smartphone-based solutions for fall detection and prevention: The FARSEEING approach. Z. für Gerontol. und Geriatr..

[18] Jara A.J., Zamora M.A., Skarmeta A.F.G. (2011). An Ambient Assisted Living Platform to Integrate Biometric Sensors to Detect Respiratory Failures for Patients with Serious Breathing Problems. International Workshop on Ambient Assisted Living.

[19] Rantz M.J., Skubic M., Koopman R.J., Phillips L., Alexander G.L., Miller S.J., Guevara R.D. Using sensor networks to detect urinary tract infections in older adults. Proceedings of the 2011 IEEE 13th International Conference on e-Health Networking, Applications and Services.

[20] Fiorini L., Cavallo F., Dario P., Eavis A., Caleb-Solly P. (2017). Unsupervised Machine Learning for Developing Personalised Behaviour Models Using Activity Data. Sensors.

[21] Ahmed N., Rafiq J.I., Islam R. (2020). Enhanced human activity recognition based on smartphone sensor data using hybridfeature selection model. Sensors.

[22] Novák M., Jakab F., Lain L. (2013). Anomaly Detection in User Daily Patterns in Smart-Home Environment. J. Sel. Areas Health Inform..

[23] Mathias S., Nayak U.S., Isaacs B. (1986). Balance in elderly patients: The “get-up and go” test. Arch. Phys. Med. Rehabil..

[24] Katz S., Ford A.B., Moskowitz R.W., Jackson B.A., Jaffe M.W. (1963). Studies of illness in the aged: The index of adl: A standardized measure of biological and psychosocial function. JAMA.

[25] Graf C. (2008). The lawton instrumental activities of daily living scale. AJN Am. J. Nurs..

[26] Folstein M.F., Folstein S.E., McHugh P.R. (1975). “Mini-mental state”: A practical method for grading the cognitive state of patients for the clinician. J. Psychiatr. Res..

[27] Nasreddine Z.S., Phillips N.A., Bédirian V., Charbonneau S., Whitehead V., Collin I., Cummings J.L., Chertkow H. (2005). The montreal cognitive assessment, moca: A brief screening tool for mild cognitive impairment. J. Am. Geriatr. Soc..

[28] Yesavage J.A., Brink T.L., Rose T.L., Lum O., Huang V., Adey M., Leirer V.O. (1983). Development and validation of a geriatric depression screening scale: A preliminary report. J. Psychiatr. Res..

[29] McCusker J., Cole M.G., Dendukuri N., Belzile E. (2004). The delirium index, a measure of the severity of delirium: New findings on reliability, validity, and responsiveness. J. Am. Geriatr. Soc..

[30] Inouye S.K., Dyck C.H.V., Alessi C.A., Balkin S., Siegal A.P., Horwitz R.I. (1990). Clarifying confusion: The confusion assessment method: A new method for detection of delirium. Ann. Intern. Med..

[31] Dahmen J., Cook D. (2019). SynSys: A synthetic data generation system for healthcare applications. Sensors.

[32] Meingast M., Roosta T., Sastry S. Security and privacy issues with health care information technology. Proceedings of the Annual International Conference of the IEEE Engineering in Medicine and Biology Society.

